# NK cell compartment in patients with coronary heart disease

**DOI:** 10.1186/1742-4933-4-3

**Published:** 2007-05-08

**Authors:** Łukasz Hak, Jolanta Myśliwska, Joanna Więckiewicz, Krzysztof Szyndler, Piotr Trzonkowski, Janusz Siebert, Andrzej Myśliwski

**Affiliations:** 1 Department of Histology and Immunology, Medical University of Gdańsk, Gdańsk, Poland; 2 Academic Clinic of Cardiosurgery, Medical University of Gdańsk, Gdańsk, Poland; 3 Department of Family Medicine, Medical University of Gdańsk, Gdańsk, Poland

## Abstract

**Background:**

Viral and bacterial infections have been considered as a risk factor for Coronary Heart Disease (CHD). NK cells, as a first line of defense against those infections, may play a role in CHD development. Thus, the main aim of our study was to determine NK cell compartment in patients with CHD undergoing coronary artery by-pass grafting.

**Results:**

Ninety three patients with CHD were included into the study; the control group consisted of 49 healthy volunteers. As compared to controls, CHD patients had lower NK cytotoxic activity. CHD group had also a decreased absolute number and percentage of total NK cells and CD3-CD56dim cytotoxic NK subset. In addition, we observed tendency toward lower percentage of the CD3-CD56bright regulatory NK subset and CD3-CD56+IFN-γ+ cells in CHD patients.

**Conclusion:**

These data indicate that CHD is associated with an impairment of NK cells compartment.

## Background

NK are a component of nonspecific immune response involved in the defense against viral and bacterial infections [1]. It is well-proved that these cells play an important regulatory role in the innate immune response. Based on the CD56 cell surface receptor density, human NK cells may be divided into two distinct subpopulations, i.e. CD56dim and CD56bright NK cells [2]. The CD56dim cells comprise about 90% of CD56 positive NK cells [3]. They exert the cytotoxic effect through perforins and granzymes [1]. These cells also possess the ability to form conjugates with appropriate target cells [4]. In contrast, the CD56bright cells are involved in the regulation of innate immune response through secretion of an array of cytokines, such as IFN-γ, TNF-β, IL-10, IL-13 and GM-CSF [5,6]. When quiescent, the CD56bright NK cells are less cytotoxic than CD56dim NK cells.

Coronary heart disease (CHD) has been regarded as a disease which aetiology might be at least in part related to chronic infections and prolonged inflammatory state [7,8]. Apart from classical risk factors such as metabolic disorders, hypertension, diabetes and age, also infections and pathogen burden have been considered as a potential risk factor for CHD. The main suspects linked to the vascular disease are wide-spread organisms such as cytomegalovirus (CMV) and *Chlamydia pneumoniae*. DNA of these pathogens was found in early atherosclerotic lesions [9,10]. Also animal models confirmed an association between infections and vascular diseases. It was found that infections with CMV, as well as with *Chlamydia pneumoniae*, accelerate development of atherosclerosis in ApoE-/- mice [11,12].

Clearly, as infections deteriorate the course of CHD and NK cells are the first line of defense against infections, there might be a link between development of CHD and NK cells. In 1997 Ogata and coworkers showed that low NK cytotoxic activity in the elderly correlates with a history of severe infections or death due to infections [13]. Moreover, the decrease in NK cell cytotoxic activity is related to susceptibility to common infectious diseases in the elderly [14]. It was also postulated that the number of NK cells correlates positively with age, which is regarded as a possible compensation for decrement of NK cell cytotoxicity during ageing [15]. Decreased NK cells cytotoxic response with age was observed by Mariani and colleagues [16]. However, when very healthy elderly people were selected the decrease in cytotoxic activity was not found [17]. Thus, it may be concluded that so called "healthy ageing" is connected with higher number of NK cells and preserved NK cell cytotoxic response.

It is also worth to mention that in the elderly, NK cells constitute the main mechanism of antiviral defense [18]. Moreover, cytotoxic activity of these cells may be modulated during infections by both bacteria and viruses [19-21]. Also inflammation affects NK cell functions; our previous studies showed that high concentration of proinflammatory cytokines correlates with low NK cytotoxic activity [22,23]. Similar data were obtained in sepsis [24].

NK activity is a key component of immune defence against obligate, intracellular bacterial and viral pathogens. Their low activity may lead to the development of persistent infections and its late consequences including enhanced risk of CHD. Not to mention that inflammation connected with CHD may further decrease NK cell functions. Thus, we aimed to characterise NK cell compartment, i.e. CD56dim/bright NK cell number and their cytotoxic activity in patients with long-lasting CHD. We found that the CHD patients had lower NK cytotoxic activity, decreased absolute number and percentage of total NK cells as well as the CD3-CD56dim cytotoxic NK subset and, CD3-CD56bright regulatory NK subset.

## Results

### Basic clinical data

As it is shown in Table 1, there were no differences in age and sex between the CHD and control groups. Not surprisingly, the percentage of people with a smoking history as well as individuals suffering from hypertension was higher in the group with coronary heart disease. Additionally, the groups differed in a spectrum of medical treatment. None of the drugs influenced significantly the examined parameters of NK activity.

**Table 1:** Basic clinical data

	***CHD patients (n = 93)***	***Control group (n = 49)***	***p***
Age (years)(median, 25th percentile/75th percentile)	63, 57/69	64, 58/73	0,43
Men (%)	56,99	42,86	0,11
Hypertension (%)	79,78	10,20	**0,0000**
Diabetes (%)	37,08	0	-
Stable angina (%)	100	-	
Smokers (%)			
Current Ex-smokers Non-smokers	15,19 56,96 27,85	10,53 39,47 50,00	**0,03**
			
NYHA, median, interquartile range	0, 0–1	-	-
CCS, median, interquartile range	2, 2–3	-	-
EuroSCORE, median, interquartile range	3, 1–4	-	-
Medicines (%)			
β-blockers	80,85	6	**0,0000**
Statins	82,98	0	-
Ca – blockers	18,08	4	0,45
Nitrates	55,32	2	**0,0000**
Diuretics	23,40	2	0,07
Spironolacton	6,38	0	-
Insulin	14,89	0	-
Oral anti-diabetics	22,34	0	-
ASA	91,49	0	-
Digoxin	0	0	-
ACE inhibitors	72,34	0	-

The results are shown as median, 25th percentile/75th percentile.

### NK cells activity

Our first approach aimed at specifying the activity of natural killer cells in the groups. We estimated it by both, NK cells cytotoxic assay and by measuring of IFN-γ intracellular production in CD3-CD56+ cells. There were significant differences in the NK cytotoxic activity and intracellular production of IFN-γ between the patients and the control group (Table 2). Natural killer cells from CHD group were less cytotoxic than those from healthy individuals. We also observed tendency towards lower production of intracellular IFN-γ by NK cells in CHD group (p = 0,07).

**Table 2:** NK cells activity

	***CHD Group n = 93***	***Control group n = 49***	***p***
The percentage of NK cells cytotoxic activity	26,83, 18,61/40,03	36,85, 24,94/43,23	**0,04**
The percentage of CD3-CD56+IFN-γ cells	4,58, 2,72/7,8	6,38, 4,03/9,83	0,07

The results are shown as median, 25th percentile/75th percentile.

All the differences were calculated by the U-Mann Whitney test.

### Circulating NK cell subsets

**Figure 1:**
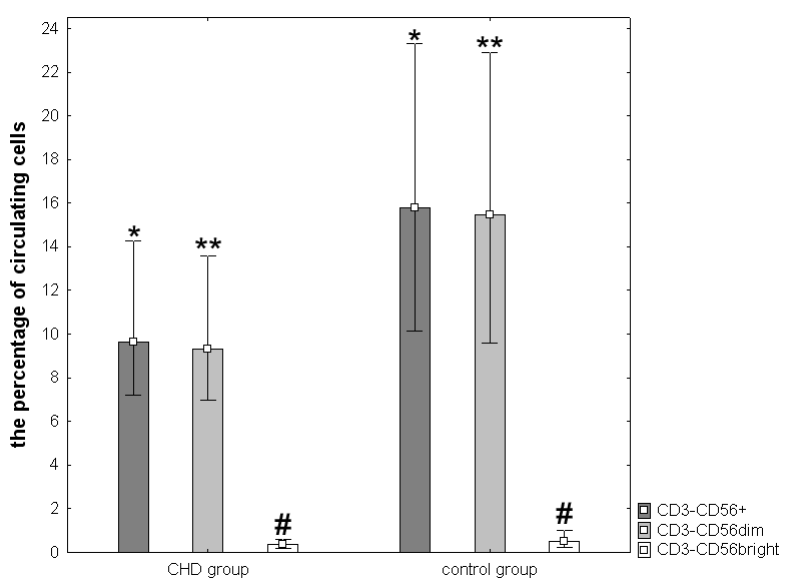
**Percentage of circulating NK cell subsets**. The results were analyzed by the U Mann Whitney test. Patients with CHD n = 93; control group n = 49. * p = 0,00003, ** p = 0,00005, # p = 0,06. The results are presented as median, 25th percentile/75th percentile.

Next we asked a question, if the lower NK activity in the patients with coronary heart disease may be connected with changes in the composition of NK compartment. To address this issue we studied changes in the number of total NK cells, the CD3-CD56bright NK regulatory cells and the CD3-CD56dim NK cytotoxic cells by flow cytometry. The percentage of circulating NK cells (CD3-CD56+), measured within lymphocyte gate, was lower in CHD patients than in the control group (p = 0,00003). Similarly, the CD3-CD56dim cells were less frequent in CHD group (p = 0,00005, Fig. 1). We found also a trend towards the difference in the percentage of CD3-CD56bright cells (p = 0,06, Fig. 1). These results were confirmed by the analysis of total number of circulating NK cells (Table 3). The absolute values of CD3-CD56+ and CD3-CD56dim cells were lower in CHD group as compared to the control group. Furthermore, the absolute number of leucocytes was higher in CHD group than in the control group.

**Table 3:** Absolute values of circulating leukocytes, lymphocytes and NK cells

***Number of cells per μL***	***CHD Group n = 93***	***Control group n = 49***	***p***
leukocytes	7300, 6120/8820	6180, 5170/7450	**0,006**
lymphocytes	1990,4, 1642,98/2463,73	1939,36, 1505,92/2633,4	0,87
CD3-CD56+ cells	200,8, 135,99/265,33	322,75, 188,26/470,68	**0,007**
CD3-CD56dim cells	191,163, 130,13/261,07	317,54, 186,17/451,15	**0,008**
CD3-CD56bright cells	6,5, 3,77/10,94	8,32, 2,46/21,08	0,35

The results are shown as median, 25th percentile/75th percentile.

All the differences were calculated by the U-Mann Whitney test.

### Smoking, medical treatment and NK cells

There was no significant effect of smoking and medical treatment on NK cells subsets and functions (p > 0,1).

## Discussion

Results of our paper revealed that patients with coronary heart disease are characterized by lower cytotoxic NK activity as compared to age-matched healthy control group. CHD patients had significantly lower absolute number and percentage of total NK cells as well as the main cytotoxic NK subset, i.e. CD56dim cells. The percentage of regulatory NK subset CD56bright was also lower in CHD patients, however, the difference did not reach statistical significance. Production of intracellular IFN-γ in CD3-CD56+ NK cells, which may be considered as a marker of NK activity, was slightly lower in CHD patients.

Our results complement and extended previous studies of Bruunsgaard [25] and Jonasson [26] who have documented NK cells impairment in cardiovascular diseases. Bruunsgaard revealed that patients with low ankle-branchial arterial pressure index, which indicates an early, symptom-free stage of atherosclerosis, are characterized by low cytotoxicity per NK cell. However, total NK cell cytotoxic activity in Bruunsgaard's study was similar to the control group as CHD patients had slightly higher number of NK cells. It is worthy to stress out that the Bruunsgaard's data illustrates the changes in NK cells compartment in an early atherosclerosis and not in fully symptomatic CHD, which can explain discrepancy between this report and our study.

Study of Jonasson [26] showed that patients with stable angina are characterized by a decrease in NK cell cytotoxic activity. Our work confirmed this finding and provided some novel data. In Jonasson's studies only the percentage of total CD3-CD56+ NK cells and its CD3-CD56dim subset were decreased in CHD patients. In contrast to our study, they did not find a difference in the absolute number of those cells. We found a trend towards lower percentage of the CD3-CD56bright cells in CHD patients in comparison to healthy controls. Furthermore, we also revealed that NK cells from CHD patients produced less IFN-γ which may explain their lower cytotoxic activity. However it has to be reminded that this data was at the edge of significance.

The reason why our study is novel comes from the fact that our experimental group is different from those previously analyzed. Bruunsgaard [25] analyzed patients with an early atherosclerosis. Jonasson studied more progressed patients but they were younger than our cohort and still they did not reach the stage requiring CABG surgery. It seems that changes in NK cell compartment are progressing with CHD development. That's why in the previous studies, which examined group of patients with early stages of CHD, impairment of NK cells compartment was not so extensive. Thus, the measuring of NK cells status may be considered as an indicator of health risk in patients with cardiovascular disorders.

In the light of the current literature the loss of NK cell functions may have an important impact on patients with CHD. The cytotoxic activity of natural killer cells may be considered as a predictor of long life and a marker of immune status. Centerians have higher NK values in relation to middle aged people and similar to the young ones [18]. Along with the cytotoxic function also the CD3-CD56dim cells, the subset with high cytotoxic properties, was found to be enlarged in the elderly [27]. Successful aging was connected with a high NK cytotoxic activity and increased number of the total NK compartment. Results of our study indicate that in patients with advanced CHD the remodeling of NK cells compartment is disadvantageous. This indicates that CHD patients are affected both by the coronary vessel disease and deficiency of NK cell activity. Searching for culprits of immunosuppression in CHD patients, the role of pathogens has to be considered. Recently, infections have been regarded as a potential risk factor for coronary heart disease. The main suspects linked to the vascular disease are herpesvirus, cytomegalovirus (CMV) and gram negative bacteria. These pathogens may depress immune system in different ways. In the early phase of infection cytomegalovirus causes release of IL-10 [28]. Similarly, production of IL-10 by monocytes is elevated during the infection with *Chlamydia pneumoniae *[29]. IL-10 is a cytokine which directly influences NK cells quenching their cytotoxic activity [30]. IL-10 counteracts IL-2 secretion, which results in decrease of NK cell function [31,32]. Moreover, an increase of the CD8+CD28-CD57+ T cells was observed during CMV long-term persistent infection [33]. The expansion of these cells was also reported in CHD patients, and the number of these cells correlated positively with anti-CMV IgG titers [34]. The CD8+CD28-CD57+ T cells are considered to be mostly CMV specific and expansion of these cells is not neutral for NK cells. In 1991 Autran and coworkers showed that the CD8+ CD57+ may have suppressive effect on NK cells functions [35]. These data indicate that an active infection with specific pathogens may affect NK cells functions. Vice versa, low NK activity may be one of the possible gates of spreading infection, and this may be a way of deepening of NK cell deficiency. Altogether, it creates a positive feedback loop leading to accelerated progress of diseases associated with chronic infections. This mechanism has a primary meaning in ageing. Ogata and colleagues studies suggested that low NK cells activity is a marker of past infections and also a predicting factor of short survival due to infections in elderly people [13,14]. Thus, low cytotoxic activity of NK cells is a factor, which connects health status of CHD patients with their susceptibility to infections.

The tendency towards decrease of the level of CD3-CD56bright cells found in our study fits with the hypothesis that NK cell functions deteriorate due to chronic inflammation. It has been suggested that CD3-CD56bright cells are very capable of migrating to the sites of local inflammation where they enhance inflammation by the stimulation of TNF-α production by monocytes [36]. Thus, decreased number of CD3-CD56bright cells in the peripheral blood of CHD patients may suggest their trafficking to the tissues affected by inflammatory process, such as artery walls, in which they accelerate unprofitable changes such as atherosclerosis.

It is also worth to mention that decrease in NK cells cytotoxic activity may be a consequence of proinflammatory effect exerted by cytokines. Our previous paper showed that older people suffering from different disorders are characterized by elevated level of these cytokines and simultaneously by low NK activity [37]. Coronary heart disease is associated with an increase of proinflammatory cytokines concentrationy [38]. Thus, an inflammation which accompanies CHD may exacerbate loss of NK cells activity.

## Conclusion

Summarizing, our data indicate that coronary heart disease is associated with an impairment of NK cells compartment.

## Methods

### Patients

Ninety three patients undergoing elective first time coronary artery by-pass grafting from the Clinic of Cardiosurgery of the Medical University of Gdańsk were selected.

Basic characteristics of the patients is shown in Table 1. Hypertension was defined as systolic blood pressure ≥ 140 mm Hg or diastolic blood pressure ≥ 90 mmHg, or patient's history of anti-hypertensive treatment verified at admission. Diabetes was defined as fasting glucose ≥ 126 mg/dl, non-fasting glucose ≥ 200 mg/dl, or patient's history of diabetes verified at admission. Coronary angiography was performed in all patients. Patients were qualified for coronary artery by-pass grafting (CABG) if they had at least one stenosis with diameter of ≥ 75% in the right coronary artery (RCA) and/or left-anterior descending artery (LAD) and/or left circumflex artery (CX). Forty nine people, of similar age, with excluded coronary heart disease were enrolled into the study as a control group. The disease was excluded on the base of physical examination, lack of clinical symptoms of CHD and normal resting and exercise-related electrocardiogram. The written informed consent was obtained from all participants. The present study was approved by the Ethics Committee of the Medical University of Gdańsk.

### Specimen collection and isolation of cells

Venous blood samples (10 ml) were collected between 9.00 and 10.00 am aseptically into the tubes with anti-coagulant (EDTA tubes Medlab, Austria) one day before a surgery.

The peripheral blood mononuclear cells (PBMC) were obtained, from venous blood, by centrifugation in Ficoll-Hypaque gradient. The PBMC were cultured in RPMI 1640 medium with 5% heat-inactivated fetal calf serum (FCS) on a plastic plate (Gibco, BRL Life Technologies, USA). After 1 h of incubation, in a humidified atmosphere containing 5% CO_2 _at 37°C, non-adherent peripheral blood lymphocytes (PBL) were collected.

### NK cytotoxic assay

Cytotoxic activity of PBL was determined in a colorimetric assay based on the measurement of lactate dyhydrogenase (LDH) activity released from the cytosol of damaged K562 target cells (human erythroleukemia cell line) into the supernatant with the use of the "Cytotoxicity Detection Kit" (Roche Diagnostics, Germany). The NK-sensitive K562 tumor line was grown in RPMI 1640 medium supplemented with 5% FCS, 100 μg/ml streptomycin, 100 U/ml penicillin (Sigma Chemical Co., USA), in a humidified atmosphere containing 5 CO_2 _at 37°C. PBL were suspended in RMPI 1640 medium supplemented with 1% FCS at the concentration of 2 × 10^5 ^cells/ml, which was the optimal concentration of effector cells (PBL) determined in pilot studies, yielding optimal levels of killing (increasing toward a plateau). Cell cultures of 100 μl PBL (2 × 10^5 ^cells/ml) and 100 μl target cells (2 × 10 ^4 ^cells/ml) in triplicates were incubated in round bottom microtiter plates for 4 h (effector-target cell ratio = 10:1) in a humidified atmosphere containing 5 CO_2 _at 37°C. The plates were then centrifuged for 10 min at 250 g, 100 μl of the supernatants were transferred to flat bottom microtiter plates, and the activity of LDH was determined using the "Cytotoxicity Detection Kit" (Roche Diagnostics, Germany). Optical density was read at 492 nm on the automated plate reader (Bio-Tek FL600, Bio-Tek Instruments, Inc. Winooski, USA). Spontaneous LDH release was determined by incubation of 100 μl target cells with 100 μl medium. The maximum release was determined by incubation of 100 μl target cells plus 100 μl medium with a final concentration of 1% Triton X-100 (Sigma Chemical Co., USA). The percentage of NK cytotoxicity, was calculated by the following formula: NK cytotoxicity (%) = [(experimental value - spontaneous release)/(maximum release - spontaneous release)] × 100 %

### Staining of NK cells

The samples of venous blood were aliquoted into plastic tubes (Falcon, Becton Dickinson Company, USA), 100 μl per tube. Cells were stained with anti-CD3 (IgG2a,κ mouse PE-Cy5, Clone: HIT3a, PharMingen, Becton Dickinson Company, USA), anti anti-CD56 (IgG1,κ mouse PE, Clone: B159, PharMingen, Becton Dickinson Company, USA) human antibodies (20 μg/test). For each set, appropriate isotypic control was done (IgG2b,κ mouse PE-Cy5, Clone: 27–35, IgG1k mouse PE, Clone: MOPC-21, PharMingen, Becton Dickinson Company, USA). After 30 min incubation in the dark at a RT samples were fixed using Immuno-prep reagents (Immunotech, USA) with Q-prep Immunology Workstation (Coulter, USA).

### Cultures of peripheral blood mononuclear cells

PBMC were obtained by centrifugation in Ficoll-Hypaque gradient and subsequently cultured on the plastic 24-well plates in triplicates, 1 × 10^6 ^cells per well, in 1 ml of RPMI containing 5% FCS. The cultures were incubated with with phorbol 12-myristate 13-acetate [PMA] for 5 hours (Sigma Chemical Co. USA) and ionomycin (Sigma Chemical Co. USA), both at final concentrations of 50 ng/ml. Two microliters of GolgiPlug (PharMingen, Becton Dickinson, USA) were added together with PMA and ionomycin to avoid a leakage of the cytokine. Control cultures were incubated for 5 hours (h) only with PMA and ionomycin

### Staining of intracellular cytokines

The PBMC cultures were vortexed and aliquoted into 12 × 75 mm plastic tubes (Falcon, Becton Dickinson, USA), 2 × 10^5 ^cells per tube. After washing of cells with staining buffer (PBS without Ca^2+ ^and Mg^2+ ^with 1% heat-inactivated foetal calf serum and 0.09% sodium azide), cells were stained with anti-CD3 (IgG2a,κ mouse PE-Cy5, Clone: HIT3a, PharMingen, Becton Dickinson Company, USA), anti anti-CD56 (IgG1,κ mouse PE, Clone: B159, PharMingen, Becton Dickinson Company, USA) human antibodies and incubated 30 min in the dark at a room temperature (RT). Then, the cells were washed and fixed in 4% paraformaldehyde (Sigma Chemical Co., USA) in PBS without Ca^2+ ^and Mg^2+^, washed and permeabilised using 0.5 ml of 0.1% saponin (in PBS without Ca^2+ ^and Mg^2+ ^with 1% heat-inactivated foetal calf serum and 0.09% sodium azide) for 20 min at a RT. Then, the cells were washed and stained with anti IFN-γ (IgG_1_,κ mouse FITC, Clone: B27, Pharmingen, BD USA) and isotype mAbs (IgG_1_, κ mouse FITC, Clone: MOPC-21, Pharmingen, BD USA) [1 μg/test] and incubated 30 min in the dark at a RT. The samples were subsequently washed and fixed in a solution of PBS containing 2% paraformaldehyde (Sigma Chemical Co., USA).

### Acquisition and analysis of flow cytometry data

Listmodes were acquired on Epics XL flow cytometer (Coulter, USA) and analyzed using Winlist, version 5.0, software. Dead cells were excluded by forward (FSC) and side (SS) angle scattered light window. The region containing lymphocytes was generated on the basis of using their forward versus right angle light scatters. A lymphocytes gate was used to measure the proportion of NK cells subsets in the sample. Typically, 10 000 events were acquired in this region. The absolute number of cells was determined by multiplying the respective percentages obtained by flow cytometry by respective absolute counts from clinical laboratory reports.

### Statistics

Data were computed using program Statistica 6.0 (Statsoft, Poland). Parametric and non-parametric distributions was assessed by W Shapiro-Wilk test. The analysis was based on non-parametric statistic U-Mann-Whitney test as indicated by data distribution.

## Competing interests

The author(s) declare that they have no competing interests.

## Authors' contributions

ŁH carried out experiments, statistical analysis and drafted the manuscript.

JM design and coordination of study and helped to draft the manuscript.

JW participated in flow cytometry analysis.

KS was responsible for CHD patients examination and selection.

PT was involved in control group selection and also participated in preparation of manuscript.

JS consulted the clinical results.

AM consulted results and manuscript preparation.

All authors read and approved the final manuscript.
